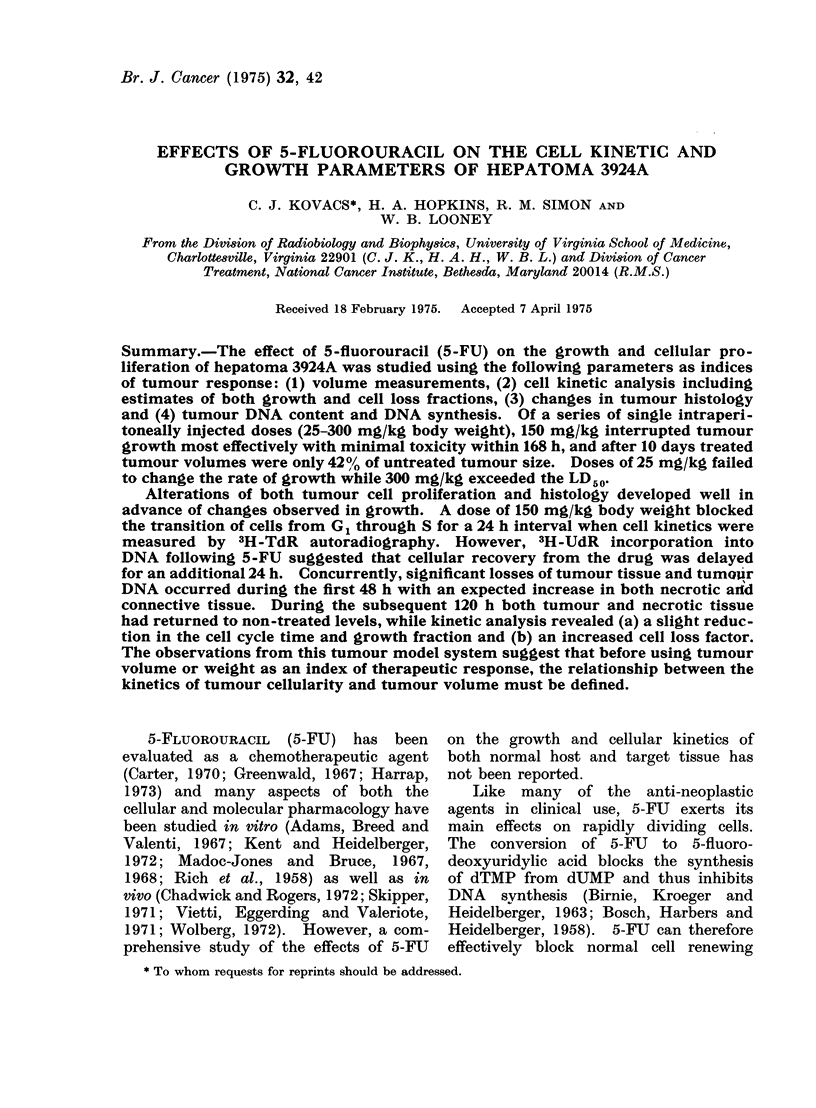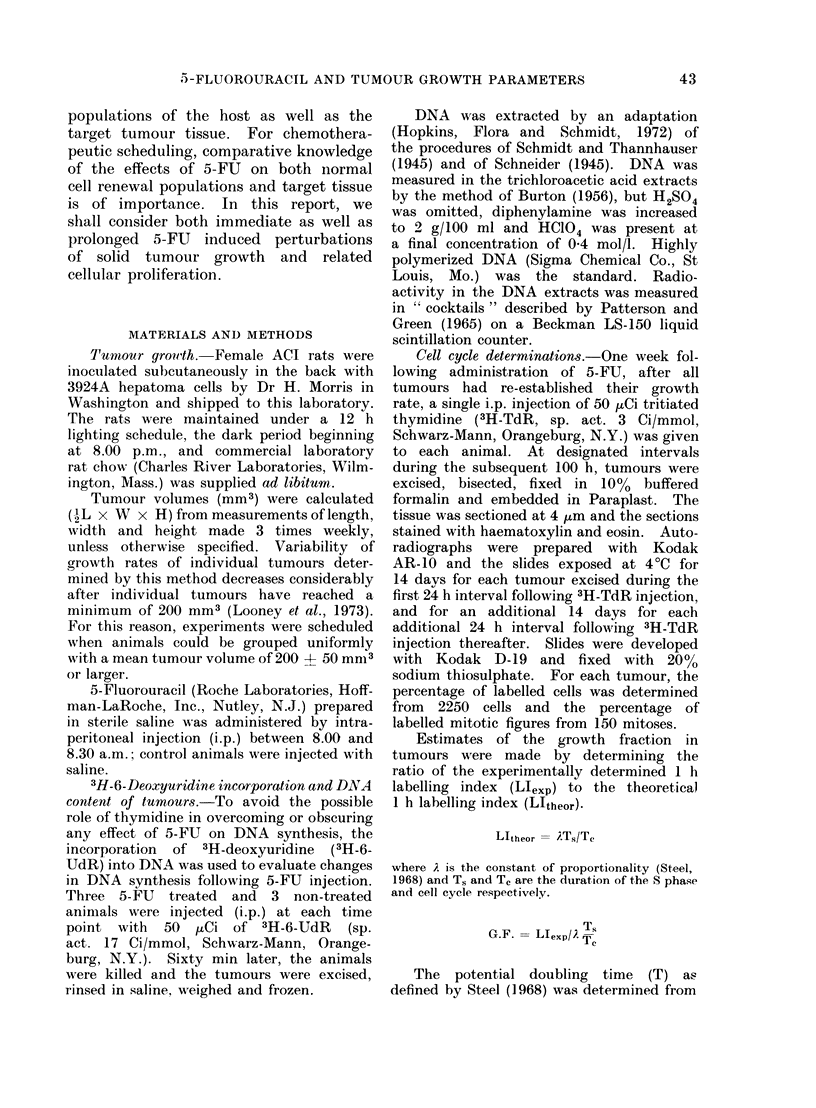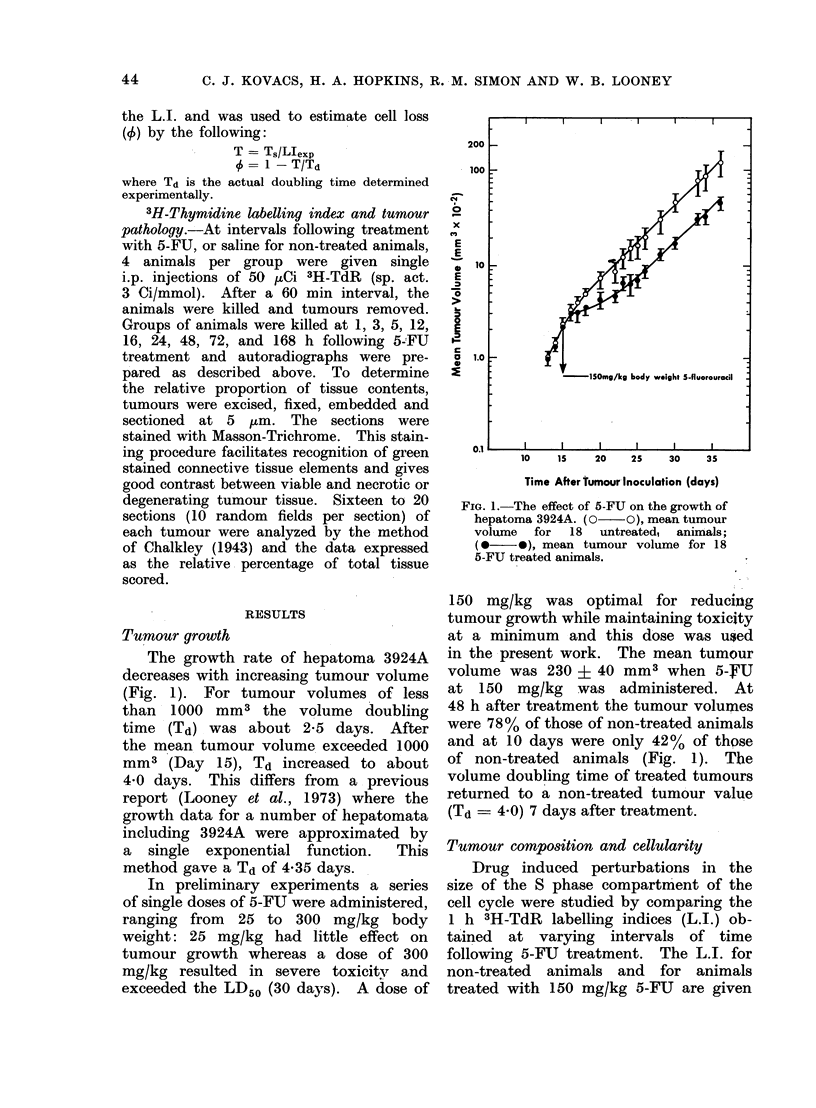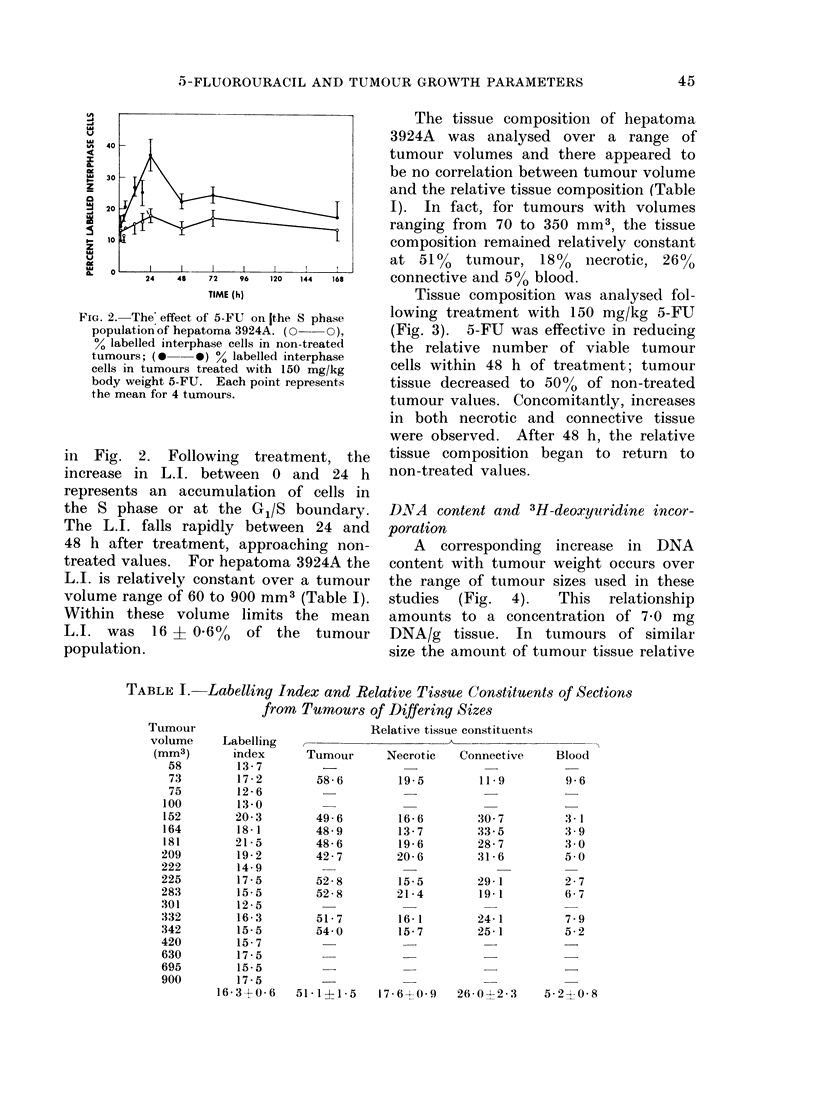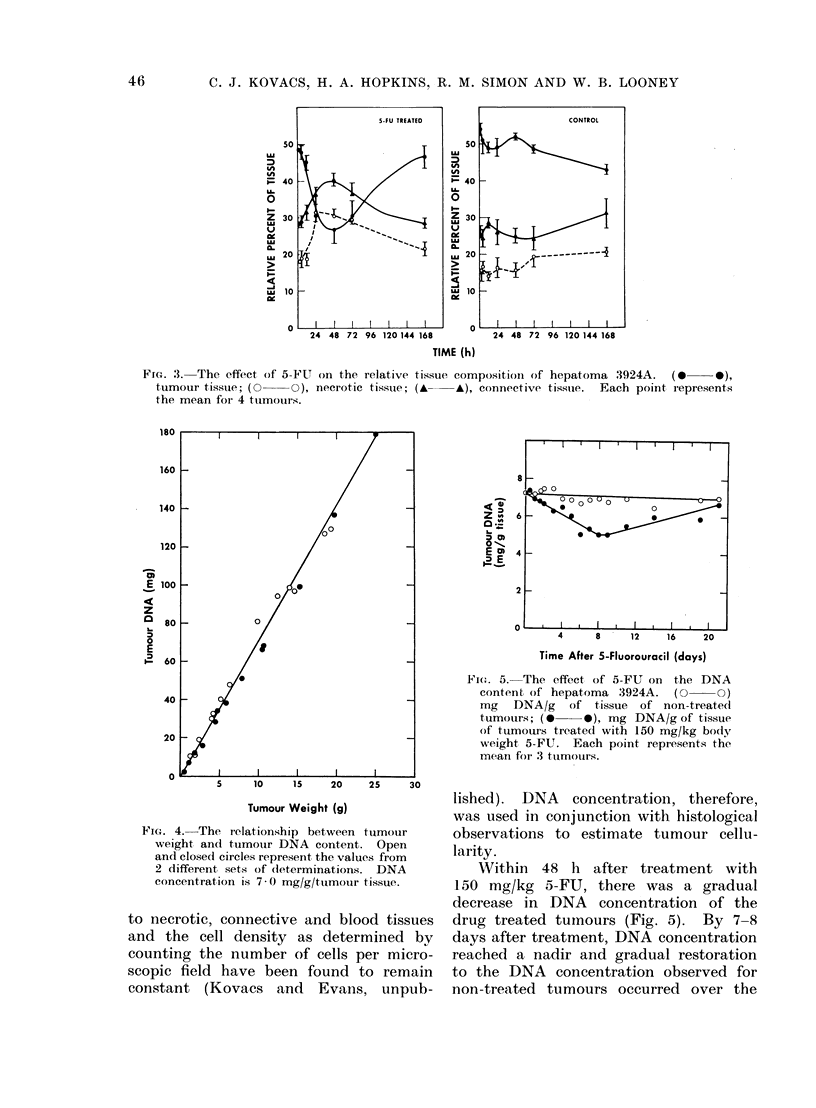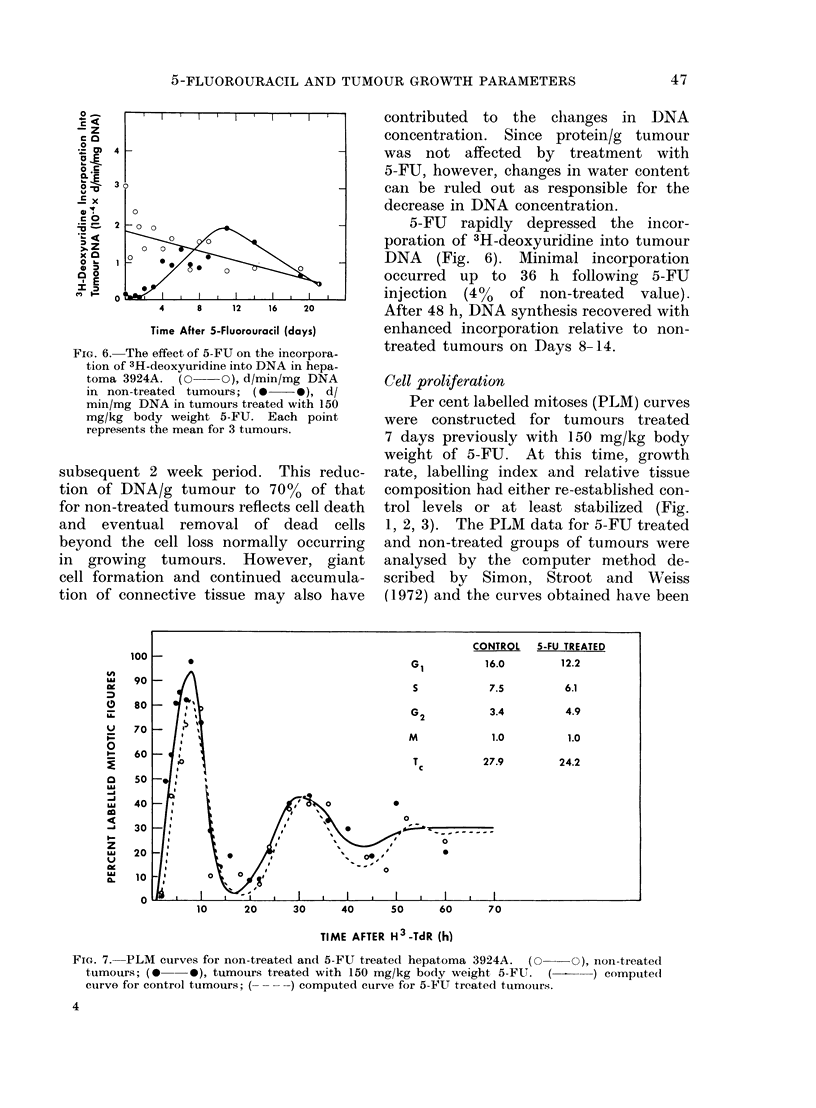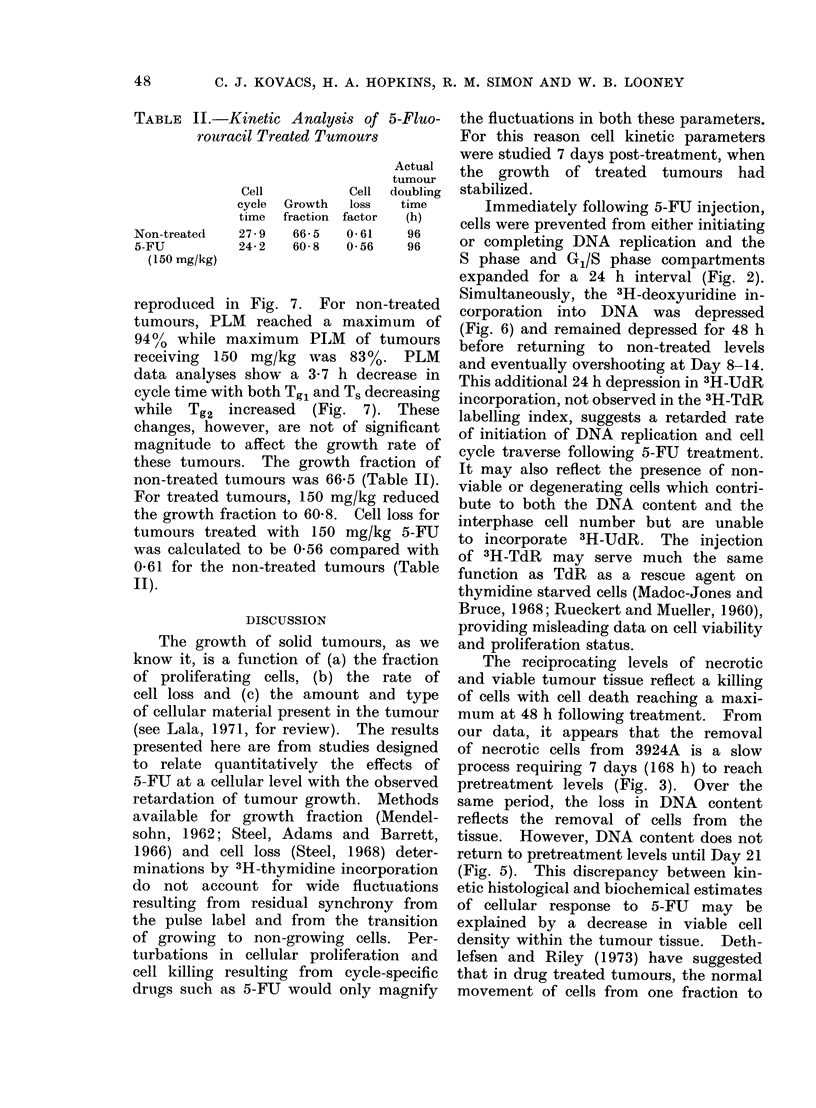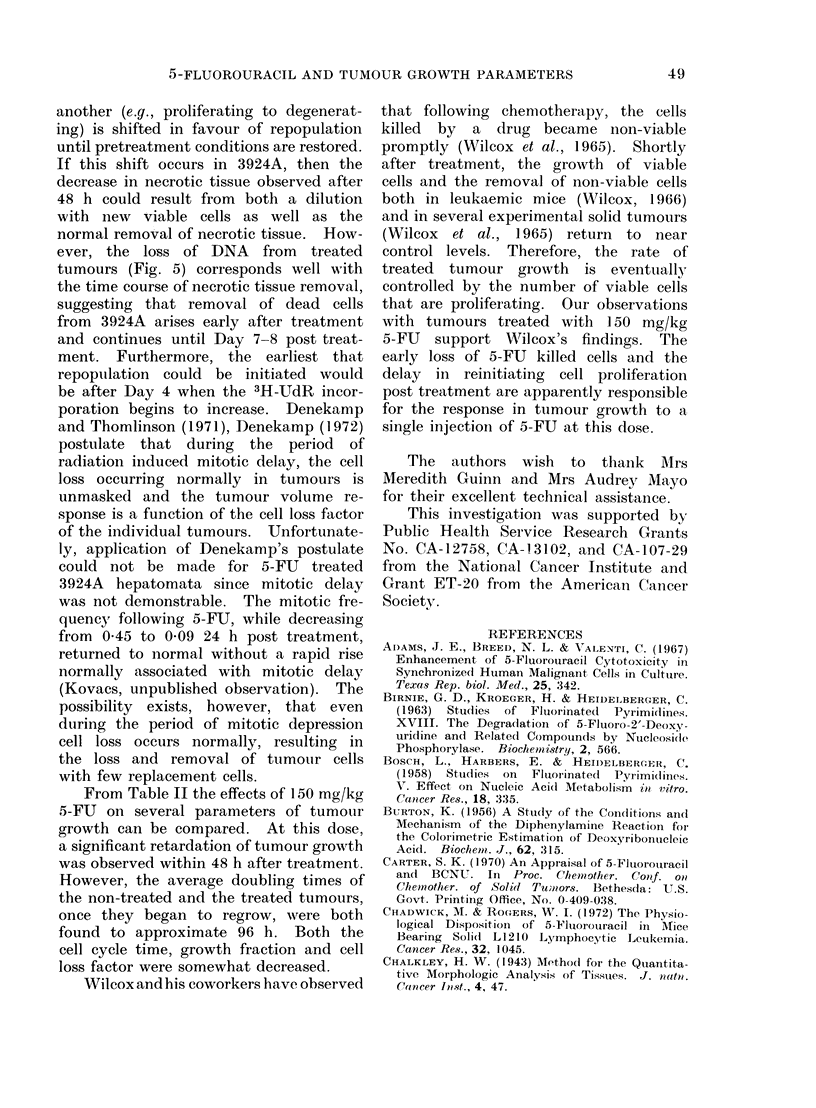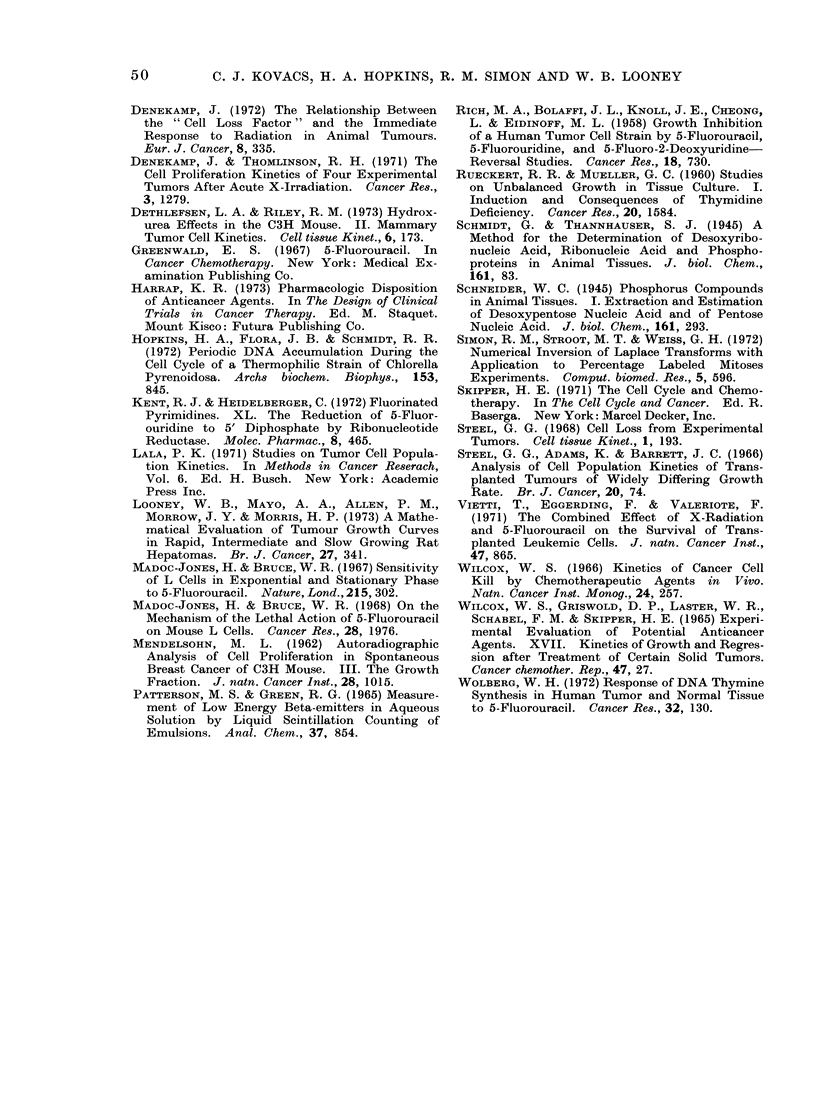# Effects of 5-fluorouracil on the cell kinetic and growth parameters of hepatoma 3924A.

**DOI:** 10.1038/bjc.1975.132

**Published:** 1975-07

**Authors:** C. J. Kovacs, H. A. Hopkins, R. M. Simon, W. B. Looney

## Abstract

The effect of 5-fluorouracil (5-FU) on the growth and cellular proliferation of hepatoma 3924A was studied using the following parameters as indices of tumour response: (1) volume measurements, (2) cell kinetic analysis including estimates of both growth and cell loss fractions, (3) changes in tumour histology and (4) tumour DNA content and DNA synthesis. Of a series of single intraperitoneally injected doses (25-300 mg/kg body weight), 150 mg/kg interrupted tumour growth most effectively with minimal toxicity within 168 h, and after 10 days treated tumour volumes were only 42% of untreated tumour size. Doses of 25 mg/kg failed to change the rate of growth while 300 mg/kg exceeded the LD50. Alterations of both tumour cell proliferation and histology developed well in advance of changes observed in growth. A dose of 150 mg/kg body weight blocked the transition of cells from G1 through S for a 24 h interval when cell kinetics were measured by 3H-TdR autoradiography. However, 3H-UdR incorporation into DNA following 5-FU suggested that cellular recovery from the drug was delayed for an additional 24 h. Concurrently, significant losses of tumour tissue and tumour DNA occurred during the first 48 h with an expected increase in both necrotic and connective tissue. During the subsequent 120 h both tumour and necrotic tissue had returned to non-treated levels, while kinetic analysis revealed (a) a slight reduction in the cell cycle time and growth fraction and (b) an increased cell loss factor. The observations from this tumour model system suggest that before using tumour volume or weight as an index of therapeutic response, the relationship between the kinetics of tumour cellularity and tumour volume must be defined.


*Br. J. Cancer* (1975) **32**, 42

### EFFECTS OF 5-FLUOROURACIL ON THE CELL KINETIC AND GROWTH PARAMETERS OF HEPATOMA 3924A

C. J. KOVACS*, H. A. HOPKINS, R. M. SIMON AND W. B. LOONEY

*From the Division of Radiobiology and Biophysics, University of Virginia School of Medicine, Charlottesville, Virginia* 22901 (*C. J. K., H. A. H., W. B. L.*) *and Division of Cancer Treatment, National Cancer Institute, Bethesda, Maryland* 20014 (*R.M.S.*)

Received 18 February 1975. Accepted 7 April 1975

**Summary.—The effect of 5-fluorouracil (5-FU) on the growth and cellular proliferation of hepatoma 3924A was studied using the following parameters as indices of tumour response: (1) volume measurements, (2) cell kinetic analysis including estimates of both growth and cell loss fractions, (3) changes in tumour histology and (4) tumour DNA content and DNA synthesis. Of a series of single intraperitoneally injected doses (25–300 mg/kg body weight), 150 mg/kg interrupted tumour growth most effectively with minimal toxicity within 168 h, and after 10 days treated tumour volumes were only 42% of untreated tumour size. Doses of 25 mg/kg failed to change the rate of growth while 300 mg/kg exceeded the $LD_{50}$.**

**Alterations of both tumour cell proliferation and histology developed well in advance of changes observed in growth. A dose of 150 mg/kg body weight blocked the transition of cells from $G_1$ through S for a 24 h interval when cell kinetics were measured by $^3$H-TdR autoradiography. However, $^3$H-UdR incorporation into DNA following 5-FU suggested that cellular recovery from the drug was delayed for an additional 24 h. Concurrently, significant losses of tumour tissue and tumour DNA occurred during the first 48 h with an expected increase in both necrotic and connective tissue. During the subsequent 120 h both tumour and necrotic tissue had returned to non-treated levels, while kinetic analysis revealed (a) a slight reduction in the cell cycle time and growth fraction and (b) an increased cell loss factor. The observations from this tumour model system suggest that before using tumour volume or weight as an index of therapeutic response, the relationship between the kinetics of tumour cellularity and tumour volume must be defined.**

5-FLUOROURACIL (5-FU) has been evaluated as a chemotherapeutic agent (Carter, 1970; Greenwald, 1967; Harrap, 1973) and many aspects of both the cellular and molecular pharmacology have been studied *in vitro* (Adams, Breed and Valenti, 1967; Kent and Heidelberger, 1972; Madoc-Jones and Bruce, 1967, 1968; Rich *et al.*, 1958) as well as *in vivo* (Chadwick and Rogers, 1972; Skipper, 1971; Vietti, Eggerding and Valeriote, 1971; Wolberg, 1972). However, a comprehensive study of the effects of 5-FU on the growth and cellular kinetics of both normal host and target tissue has not been reported.

Like many of the anti-neoplastic agents in clinical use, 5-FU exerts its main effects on rapidly dividing cells. The conversion of 5-FU to 5-fluorodeoxyuridylic acid blocks the synthesis of dTMP from dUMP and thus inhibits DNA synthesis (Birnie, Kroeger and Heidelberger, 1963; Bosch, Harbers and Heidelberger, 1958). 5-FU can therefore effectively block normal cell renewing

* To whom requests for reprints should be addressed.

populations of the host as well as the target tumour tissue. For chemotherapeutic scheduling, comparative knowledge of the effects of 5-FU on both normal cell renewal populations and target tissue is of importance. In this report, we shall consider both immediate as well as prolonged 5-FU induced perturbations of solid tumour growth and related cellular proliferation.

### MATERIALS AND METHODS

*Tumour growth.*—Female ACI rats were inoculated subcutaneously in the back with 3924A hepatoma cells by Dr H. Morris in Washington and shipped to this laboratory. The rats were maintained under a 12 h lighting schedule, the dark period beginning at 8.00 p.m., and commercial laboratory rat chow (Charles River Laboratories, Wilmington, Mass.) was supplied *ad libitum.*

Tumour volumes ($mm^3$) were calculated ($\frac{1}{2}L \times W \times H$) from measurements of length, width and height made 3 times weekly, unless otherwise specified. Variability of growth rates of individual tumours determined by this method decreases considerably after individual tumours have reached a minimum of 200 $mm^3$ (Looney *et al.*, 1973). For this reason, experiments were scheduled when animals could be grouped uniformly with a mean tumour volume of 200 $\pm$ 50 $mm^3$ or larger.

5-Fluorouracil (Roche Laboratories, Hoffman-LaRoche, Inc., Nutley, N.J.) prepared in sterile saline was administered by intraperitoneal injection (i.p.) between 8.00 and 8.30 a.m.; control animals were injected with saline.

*$^3$H-6-Deoxyuridine incorporation and DNA content of tumours.*—To avoid the possible role of thymidine in overcoming or obscuring any effect of 5-FU on DNA synthesis, the incorporation of $^3$H-deoxyuridine ($^3$H-6-UdR) into DNA was used to evaluate changes in DNA synthesis following 5-FU injection. Three 5-FU treated and 3 non-treated animals were injected (i.p.) at each time point with 50 $\mu$Ci of $^3$H-6-UdR (sp. act. 17 Ci/mmol, Schwarz-Mann, Orangeburg, N.Y.). Sixty min later, the animals were killed and the tumours were excised, rinsed in saline, weighed and frozen.

DNA was extracted by an adaptation (Hopkins, Flora and Schmidt, 1972) of the procedures of Schmidt and Thannhauser (1945) and of Schneider (1945). DNA was measured in the trichloroacetic acid extracts by the method of Burton (1956), but $H_2SO_4$ was omitted, diphenylamine was increased to 2 g/100 ml and $HClO_4$ was present at a final concentration of 0·4 mol/l. Highly polymerized DNA (Sigma Chemical Co., St Louis, Mo.) was the standard. Radioactivity in the DNA extracts was measured in "cocktails" described by Patterson and Green (1965) on a Beckman LS-150 liquid scintillation counter.

*Cell cycle determinations.*—One week following administration of 5-FU, after all tumours had re-established their growth rate, a single i.p. injection of 50 $\mu$Ci tritiated thymidine ($^3$H-TdR, sp. act. 3 Ci/mmol, Schwarz-Mann, Orangeburg, N.Y.) was given to each animal. At designated intervals during the subsequent 100 h, tumours were excised, bisected, fixed in 10% buffered formalin and embedded in Paraplast. The tissue was sectioned at 4 $\mu$m and the sections stained with haematoxylin and eosin. Autoradiographs were prepared with Kodak AR-10 and the slides exposed at 4°C for 14 days for each tumour excised during the first 24 h interval following $^3$H-TdR injection, and for an additional 14 days for each additional 24 h interval following $^3$H-TdR injection thereafter. Slides were developed with Kodak D-19 and fixed with 20% sodium thiosulphate. For each tumour, the percentage of labelled cells was determined from 2250 cells and the percentage of labelled mitotic figures from 150 mitoses.

Estimates of the growth fraction in tumours were made by determining the ratio of the experimentally determined 1 h labelling index ($LI_{exp}$) to the theoretical 1 h labelling index ($LI_{theor}$).

$$LI_{theor} = \lambda T_S/T_C$$

where $\lambda$ is the constant of proportionality (Steel, 1968) and $T_S$ and $T_C$ are the duration of the S phase and cell cycle respectively.

$$G.F. = LI_{exp}/\lambda \frac{T_S}{T_C}$$

The potential doubling time (T) as defined by Steel (1968) was determined from the L.I. and was used to estimate cell loss ($\phi$) by the following:

$$T = T_s/LI_{exp}$$
$$\phi = 1 - T/T_d$$

where $T_d$ is the actual doubling time determined experimentally.

*$^3$H-Thymidine labelling index and tumour pathology.*—At intervals following treatment with 5-FU, or saline for non-treated animals, 4 animals per group were given single i.p. injections of 50 $\mu$Ci $^3$H-TdR (sp. act. 3 Ci/mmol). After a 60 min interval, the animals were killed and tumours removed. Groups of animals were killed at 1, 3, 5, 12, 16, 24, 48, 72, and 168 h following 5-FU treatment and autoradiographs were prepared as described above. To determine the relative proportion of tissue contents, tumours were excised, fixed, embedded and sectioned at 5 $\mu$m. The sections were stained with Masson-Trichrome. This staining procedure facilitates recognition of green stained connective tissue elements and gives good contrast between viable and necrotic or degenerating tumour tissue. Sixteen to 20 sections (10 random fields per section) of each tumour were analyzed by the method of Chalkley (1943) and the data expressed as the relative percentage of total tissue scored.

### RESULTS

*Tumour growth*

The growth rate of hepatoma 3924A decreases with increasing tumour volume (Fig. 1). For tumour volumes of less than 1000 mm$^3$ the volume doubling time ($T_d$) was about 2·5 days. After the mean tumour volume exceeded 1000 mm$^3$ (Day 15), $T_d$ increased to about 4·0 days. This differs from a previous report (Looney *et al.*, 1973) where the growth data for a number of hepatomata including 3924A were approximated by a single exponential function. This method gave a $T_d$ of 4·35 days.

In preliminary experiments a series of single doses of 5-FU were administered, ranging from 25 to 300 mg/kg body weight: 25 mg/kg had little effect on tumour growth whereas a dose of 300 mg/kg resulted in severe toxicity and exceeded the $LD_{50}$ (30 days). A dose of

FIG. 1.—The effect of 5-FU on the growth of hepatoma 3924A. (○——○), mean tumour volume for 18 untreated, animals; (●——●), mean tumour volume for 18 5-FU treated animals.

150 mg/kg was optimal for reducing tumour growth while maintaining toxicity at a minimum and this dose was used in the present work. The mean tumour volume was 230 $\pm$ 40 mm$^3$ when 5-FU at 150 mg/kg was administered. At 48 h after treatment the tumour volumes were 78% of those of non-treated animals and at 10 days were only 42% of those of non-treated animals (Fig. 1). The volume doubling time of treated tumours returned to a non-treated tumour value ($T_d$ = 4·0) 7 days after treatment.

*Tumour composition and cellularity*

Drug induced perturbations in the size of the S phase compartment of the cell cycle were studied by comparing the 1 h $^3$H-TdR labelling indices (L.I.) obtained at varying intervals of time following 5-FU treatment. The L.I. for non-treated animals and for animals treated with 150 mg/kg 5-FU are given

FIG. 2.—The effect of 5-FU on the S phase population of hepatoma 3924A. (○——○), % labelled interphase cells in non-treated tumours; (●——●) % labelled interphase cells in tumours treated with 150 mg/kg body weight 5-FU. Each point represents the mean for 4 tumours.

in Fig. 2. Following treatment, the increase in L.I. between 0 and 24 h represents an accumulation of cells in the S phase or at the $G_1/S$ boundary. The L.I. falls rapidly between 24 and 48 h after treatment, approaching non-treated values. For hepatoma 3924A the L.I. is relatively constant over a tumour volume range of 60 to 900 $mm^3$ (Table I). Within these volume limits the mean L.I. was $16 \pm 0{\cdot}6\%$ of the tumour population.

The tissue composition of hepatoma 3924A was analysed over a range of tumour volumes and there appeared to be no correlation between tumour volume and the relative tissue composition (Table I). In fact, for tumours with volumes ranging from 70 to 350 $mm^3$, the tissue composition remained relatively constant at 51% tumour, 18% necrotic, 26% connective and 5% blood.

Tissue composition was analysed following treatment with 150 mg/kg 5-FU (Fig. 3). 5-FU was effective in reducing the relative number of viable tumour cells within 48 h of treatment; tumour tissue decreased to 50% of non-treated tumour values. Concomitantly, increases in both necrotic and connective tissue were observed. After 48 h, the relative tissue composition began to return to non-treated values.

*DNA content and $^3H$-deoxyuridine incorporation*

A corresponding increase in DNA content with tumour weight occurs over the range of tumour sizes used in these studies (Fig. 4). This relationship amounts to a concentration of 7·0 mg DNA/g tissue. In tumours of similar size the amount of tumour tissue relative

TABLE I.—*Labelling Index and Relative Tissue Constituents of Sections from Tumours of Differing Sizes*

| Tumour volume ($mm^3$) | Labelling index | Relative tissue constituents | | | |
|---|---|---|---|---|---|
| | | Tumour | Necrotic | Connective | Blood |
| 58 | 13·7 | — | — | — | — |
| 73 | 17·2 | 58·6 | 19·5 | 11·9 | 9·6 |
| 75 | 12·6 | — | — | — | — |
| 100 | 13·0 | — | — | — | — |
| 152 | 20·3 | 49·6 | 16·6 | 30·7 | 3·1 |
| 164 | 18·1 | 48·9 | 13·7 | 33·5 | 3·9 |
| 181 | 21·5 | 48·6 | 19·6 | 28·7 | 3·0 |
| 209 | 19·2 | 42·7 | 20·6 | 31·6 | 5·0 |
| 222 | 14·9 | — | — | — | — |
| 225 | 17·5 | 52·8 | 15·5 | 29·1 | 2·7 |
| 283 | 15·5 | 52·8 | 21·4 | 19·1 | 6·7 |
| 301 | 12·5 | — | — | — | — |
| 332 | 16·3 | 51·7 | 16·1 | 24·1 | 7·9 |
| 342 | 15·5 | 54·0 | 15·7 | 25·1 | 5·2 |
| 420 | 15·7 | — | — | — | — |
| 630 | 17·5 | — | — | — | — |
| 695 | 15·5 | — | — | — | — |
| 900 | 17·5 | — | — | — | — |
| | 16·3±0·6 | 51·1±1·5 | 17·6±0·9 | 26·0±2·3 | 5·2±0·8 |

FIG. 3.—The effect of 5-FU on the relative tissue composition of hepatoma 3924A. (●——●), tumour tissue; (○——○), necrotic tissue; (▲——▲), connective tissue. Each point represents the mean for 4 tumours.

FIG. 4.—The relationship between tumour weight and tumour DNA content. Open and closed circles represent the values from 2 different sets of determinations. DNA concentration is 7·0 mg/g/tumour tissue.

FIG. 5.—The effect of 5-FU on the DNA content of hepatoma 3924A. (○——○) mg DNA/g of tissue of non-treated tumours; (●——●), mg DNA/g of tissue of tumours treated with 150 mg/kg body weight 5-FU. Each point represents the mean for 3 tumours.

to necrotic, connective and blood tissues and the cell density as determined by counting the number of cells per microscopic field have been found to remain constant (Kovacs and Evans, unpublished). DNA concentration, therefore, was used in conjunction with histological observations to estimate tumour cellularity.

Within 48 h after treatment with 150 mg/kg 5-FU, there was a gradual decrease in DNA concentration of the drug treated tumours (Fig. 5). By 7–8 days after treatment, DNA concentration reached a nadir and gradual restoration to the DNA concentration observed for non-treated tumours occurred over the

Fig. 6.—The effect of 5-FU on the incorporation of $^{3}$H-deoxyuridine into DNA in hepatoma 3924A. (○——○), d/min/mg DNA in non-treated tumours; (●——●), d/min/mg DNA in tumours treated with 150 mg/kg body weight 5-FU. Each point represents the mean for 3 tumours.

subsequent 2 week period. This reduction of DNA/g tumour to 70% of that for non-treated tumours reflects cell death and eventual removal of dead cells beyond the cell loss normally occurring in growing tumours. However, giant cell formation and continued accumulation of connective tissue may also have contributed to the changes in DNA concentration. Since protein/g tumour was not affected by treatment with 5-FU, however, changes in water content can be ruled out as responsible for the decrease in DNA concentration.

5-FU rapidly depressed the incorporation of $^{3}$H-deoxyuridine into tumour DNA (Fig. 6). Minimal incorporation occurred up to 36 h following 5-FU injection (4% of non-treated value). After 48 h, DNA synthesis recovered with enhanced incorporation relative to non-treated tumours on Days 8–14.

*Cell proliferation*

Per cent labelled mitoses (PLM) curves were constructed for tumours treated 7 days previously with 150 mg/kg body weight of 5-FU. At this time, growth rate, labelling index and relative tissue composition had either re-established control levels or at least stabilized (Fig. 1, 2, 3). The PLM data for 5-FU treated and non-treated groups of tumours were analysed by the computer method described by Simon, Stroot and Weiss (1972) and the curves obtained have been

Fig. 7.—PLM curves for non-treated and 5-FU treated hepatoma 3924A. (○——○), non-treated tumours; (●——●), tumours treated with 150 mg/kg body weight 5-FU. (——) computed curve for control tumours; (– – – –) computed curve for 5-FU treated tumours.

TABLE II.—*Kinetic Analysis of 5-Fluorouracil Treated Tumours*

| | Cell cycle time | Growth fraction | Cell loss factor | Actual tumour doubling time (h) |
|---|---|---|---|---|
| Non-treated | 27·9 | 66·5 | 0·61 | 96 |
| 5-FU (150 mg/kg) | 24·2 | 60·8 | 0·56 | 96 |

reproduced in Fig. 7. For non-treated tumours, PLM reached a maximum of 94% while maximum PLM of tumours receiving 150 mg/kg was 83%. PLM data analyses show a 3·7 h decrease in cycle time with both $T_{g_1}$ and $T_s$ decreasing while $T_{g_2}$ increased (Fig. 7). These changes, however, are not of significant magnitude to affect the growth rate of these tumours. The growth fraction of non-treated tumours was 66·5 (Table II). For treated tumours, 150 mg/kg reduced the growth fraction to 60·8. Cell loss for tumours treated with 150 mg/kg 5-FU was calculated to be 0·56 compared with 0·61 for the non-treated tumours (Table II).

### DISCUSSION

The growth of solid tumours, as we know it, is a function of (a) the fraction of proliferating cells, (b) the rate of cell loss and (c) the amount and type of cellular material present in the tumour (see Lala, 1971, for review). The results presented here are from studies designed to relate quantitatively the effects of 5-FU at a cellular level with the observed retardation of tumour growth. Methods available for growth fraction (Mendelsohn, 1962; Steel, Adams and Barrett, 1966) and cell loss (Steel, 1968) determinations by $^3$H-thymidine incorporation do not account for wide fluctuations resulting from residual synchrony from the pulse label and from the transition of growing to non-growing cells. Perturbations in cellular proliferation and cell killing resulting from cycle-specific drugs such as 5-FU would only magnify the fluctuations in both these parameters. For this reason cell kinetic parameters were studied 7 days post-treatment, when the growth of treated tumours had stabilized.

Immediately following 5-FU injection, cells were prevented from either initiating or completing DNA replication and the S phase and $G_1$/S phase compartments expanded for a 24 h interval (Fig. 2). Simultaneously, the $^3$H-deoxyuridine incorporation into DNA was depressed (Fig. 6) and remained depressed for 48 h before returning to non-treated levels and eventually overshooting at Day 8–14. This additional 24 h depression in $^3$H-UdR incorporation, not observed in the $^3$H-TdR labelling index, suggests a retarded rate of initiation of DNA replication and cell cycle traverse following 5-FU treatment. It may also reflect the presence of non-viable or degenerating cells which contribute to both the DNA content and the interphase cell number but are unable to incorporate $^3$H-UdR. The injection of $^3$H-TdR may serve much the same function as TdR as a rescue agent on thymidine starved cells (Madoc-Jones and Bruce, 1968; Rueckert and Mueller, 1960), providing misleading data on cell viability and proliferation status.

The reciprocating levels of necrotic and viable tumour tissue reflect a killing of cells with cell death reaching a maximum at 48 h following treatment. From our data, it appears that the removal of necrotic cells from 3924A is a slow process requiring 7 days (168 h) to reach pretreatment levels (Fig. 3). Over the same period, the loss in DNA content reflects the removal of cells from the tissue. However, DNA content does not return to pretreatment levels until Day 21 (Fig. 5). This discrepancy between kinetic histological and biochemical estimates of cellular response to 5-FU may be explained by a decrease in viable cell density within the tumour tissue. Dethlefsen and Riley (1973) have suggested that in drug treated tumours, the normal movement of cells from one fraction to another (*e.g.*, proliferating to degenerating) is shifted in favour of repopulation until pretreatment conditions are restored. If this shift occurs in 3924A, then the decrease in necrotic tissue observed after 48 h could result from both a dilution with new viable cells as well as the normal removal of necrotic tissue. However, the loss of DNA from treated tumours (Fig. 5) corresponds well with the time course of necrotic tissue removal, suggesting that removal of dead cells from 3924A arises early after treatment and continues until Day 7–8 post treatment. Furthermore, the earliest that repopulation could be initiated would be after Day 4 when the $^{3}$H-UdR incorporation begins to increase. Denekamp and Thomlinson (1971), Denekamp (1972) postulate that during the period of radiation induced mitotic delay, the cell loss occurring normally in tumours is unmasked and the tumour volume response is a function of the cell loss factor of the individual tumours. Unfortunately, application of Denekamp's postulate could not be made for 5-FU treated 3924A hepatomata since mitotic delay was not demonstrable. The mitotic frequency following 5-FU, while decreasing from 0·45 to 0·09 24 h post treatment, returned to normal without a rapid rise normally associated with mitotic delay (Kovacs, unpublished observation). The possibility exists, however, that even during the period of mitotic depression cell loss occurs normally, resulting in the loss and removal of tumour cells with few replacement cells.

From Table II the effects of 150 mg/kg 5-FU on several parameters of tumour growth can be compared. At this dose, a significant retardation of tumour growth was observed within 48 h after treatment. However, the average doubling times of the non-treated and the treated tumours, once they began to regrow, were both found to approximate 96 h. Both the cell cycle time, growth fraction and cell loss factor were somewhat decreased.

Wilcox and his coworkers have observed that following chemotherapy, the cells killed by a drug became non-viable promptly (Wilcox *et al.*, 1965). Shortly after treatment, the growth of viable cells and the removal of non-viable cells both in leukaemic mice (Wilcox, 1966) and in several experimental solid tumours (Wilcox *et al.*, 1965) return to near control levels. Therefore, the rate of treated tumour growth is eventually controlled by the number of viable cells that are proliferating. Our observations with tumours treated with 150 mg/kg 5-FU support Wilcox's findings. The early loss of 5-FU killed cells and the delay in reinitiating cell proliferation post treatment are apparently responsible for the response in tumour growth to a single injection of 5-FU at this dose.

The authors wish to thank Mrs Meredith Guinn and Mrs Audrey Mayo for their excellent technical assistance.

This investigation was supported by Public Health Service Research Grants No. CA-12758, CA-13102, and CA-107-29 from the National Cancer Institute and Grant ET-20 from the American Cancer Society.

### REFERENCES

ADAMS, J. E., BREED, N. L. & VALENTI, C. (1967) Enhancement of 5-Fluorouracil Cytotoxicity in Synchronized Human Malignant Cells in Culture. *Texas Rep. biol. Med.*, **25**, 342.

BIRNIE, G. D., KROEGER, H. & HEIDELBERGER, C. (1963) Studies of Fluorinated Pyrimidines. XVIII. The Degradation of 5-Fluoro-2′-Deoxyuridine and Related Compounds by Nucleoside Phosphorylase. *Biochemistry*, **2**, 566.

BOSCH, L., HARBERS, E. & HEIDELBERGER, C. (1958) Studies on Fluorinated Pyrimidines. V. Effect on Nucleic Acid Metabolism *in vitro*. *Cancer Res.*, **18**, 335.

BURTON, K. (1956) A Study of the Conditions and Mechanism of the Diphenylamine Reaction for the Colorimetric Estimation of Deoxyribonucleic Acid. *Biochem. J.*, **62**, 315.

CARTER, S. K. (1970) An Appraisal of 5-Fluorouracil and BCNU. In *Proc. Chemother. Conf. on Chemother. of Solid Tumors*. Bethesda: U.S. Govt. Printing Office, No. 0-409-038.

CHADWICK, M. & ROGERS, W. I. (1972) The Physiological Disposition of 5-Fluorouracil in Mice Bearing Solid L1210 Lymphocytic Leukemia. *Cancer Res.*, **32**, 1045.

CHALKLEY, H. W. (1943) Method for the Quantitative Morphologic Analysis of Tissues. *J. natn. Cancer Inst.*, **4**, 47.

DENEKAMP, J. (1972) The Relationship Between the "Cell Loss Factor" and the Immediate Response to Radiation in Animal Tumours. *Eur. J. Cancer*, **8**, 335.

DENEKAMP, J. & THOMLINSON, R. H. (1971) The Cell Proliferation Kinetics of Four Experimental Tumors After Acute X-Irradiation. *Cancer Res.*, **3**, 1279.

DETHLEFSEN, L. A. & RILEY, R. M. (1973) Hydroxurea Effects in the C3H Mouse. II. Mammary Tumor Cell Kinetics. *Cell tissue Kinet.*, **6**, 173.

GREENWALD, E. S. (1967) 5-Fluorouracil. In *Cancer Chemotherapy*. New York: Medical Examination Publishing Co.

HARRAP, K. R. (1973) Pharmacologic Disposition of Anticancer Agents. In *The Design of Clinical Trials in Cancer Therapy*. Ed. M. Staquet. Mount Kisco: Futura Publishing Co.

HOPKINS, H. A., FLORA, J. B. & SCHMIDT, R. R. (1972) Periodic DNA Accumulation During the Cell Cycle of a Thermophilic Strain of Chlorella Pyrenoidosa. *Archs biochem. Biophys.*, **153**, 845.

KENT, R. J. & HEIDELBERGER, C. (1972) Fluorinated Pyrimidines. XL. The Reduction of 5-Fluorouridine to 5′ Diphosphate by Ribonucleotide Reductase. *Molec. Pharmac.*, **8**, 465.

LALA, P. K. (1971) Studies on Tumor Cell Population Kinetics. In *Methods in Cancer Reserach*, Vol. 6. Ed. H. Busch. New York: Academic Press Inc.

LOONEY, W. B., MAYO, A. A., ALLEN, P. M., MORROW, J. Y. & MORRIS, H. P. (1973) A Mathematical Evaluation of Tumour Growth Curves in Rapid, Intermediate and Slow Growing Rat Hepatomas. *Br. J. Cancer*, **27**, 341.

MADOC-JONES, H. & BRUCE, W. R. (1967) Sensitivity of L Cells in Exponential and Stationary Phase to 5-Fluorouracil. *Nature, Lond.*, **215**, 302.

MADOC-JONES, H. & BRUCE, W. R. (1968) On the Mechanism of the Lethal Action of 5-Fluorouracil on Mouse L Cells. *Cancer Res.*, **28**, 1976.

MENDELSOHN, M. L. (1962) Autoradiographic Analysis of Cell Proliferation in Spontaneous Breast Cancer of C3H Mouse. III. The Growth Fraction. *J. natn. Cancer Inst.*, **28**, 1015.

PATTERSON, M. S. & GREEN, R. G. (1965) Measurement of Low Energy Beta-emitters in Aqueous Solution by Liquid Scintillation Counting of Emulsions. *Anal. Chem.*, **37**, 854.

RICH, M. A., BOLAFFI, J. L., KNOLL, J. E., CHEONG, L. & EIDINOFF, M. L. (1958) Growth Inhibition of a Human Tumor Cell Strain by 5-Fluorouracil, 5-Fluorouridine, and 5-Fluoro-2-Deoxyuridine—Reversal Studies. *Cancer Res.*, **18**, 730.

RUECKERT, R. R. & MUELLER, G. C. (1960) Studies on Unbalanced Growth in Tissue Culture. I. Induction and Consequences of Thymidine Deficiency. *Cancer Res.*, **20**, 1584.

SCHMIDT, G. & THANNHAUSER, S. J. (1945) A Method for the Determination of Desoxyribonucleic Acid, Ribonucleic Acid and Phosphoproteins in Animal Tissues. *J. biol. Chem.*, **161**, 83.

SCHNEIDER, W. C. (1945) Phosphorus Compounds in Animal Tissues. I. Extraction and Estimation of Desoxypentose Nucleic Acid and of Pentose Nucleic Acid. *J. biol. Chem.*, **161**, 293.

SIMON, R. M., STROOT, M. T. & WEISS, G. H. (1972) Numerical Inversion of Laplace Transforms with Application to Percentage Labeled Mitoses Experiments. *Comput. biomed. Res.*, **5**, 596.

SKIPPER, H. E. (1971) The Cell Cycle and Chemotherapy. In *The Cell Cycle and Cancer*. Ed. R. Baserga. New York: Marcel Decker, Inc.

STEEL, G. G. (1968) Cell Loss from Experimental Tumors. *Cell tissue Kinet.*, **1**, 193.

STEEL, G. G., ADAMS, K. & BARRETT, J. C. (1966) Analysis of Cell Population Kinetics of Transplanted Tumours of Widely Differing Growth Rate. *Br. J. Cancer*, **20**, 74.

VIETTI, T., EGGERDING, F. & VALERIOTE, F. (1971) The Combined Effect of X-Radiation and 5-Fluorouracil on the Survival of Transplanted Leukemic Cells. *J. natn. Cancer Inst.*, **47**, 865.

WILCOX, W. S. (1966) Kinetics of Cancer Cell Kill by Chemotherapeutic Agents *in Vivo*. *Natn. Cancer Inst. Monog.*, **24**, 257.

WILCOX, W. S., GRISWOLD, D. P., LASTER, W. R., SCHABEL, F. M. & SKIPPER, H. E. (1965) Experimental Evaluation of Potential Anticancer Agents. XVII. Kinetics of Growth and Regression after Treatment of Certain Solid Tumors. *Cancer chemother. Rep.*, **47**, 27.

WOLBERG, W. H. (1972) Response of DNA Thymine Synthesis in Human Tumor and Normal Tissue to 5-Fluorouracil. *Cancer Res.*, **32**, 130.